# Characterization and preliminary heterosis evaluation of novel wheat genetically divergent populations

**DOI:** 10.3389/fpls.2026.1824151

**Published:** 2026-06-22

**Authors:** Ziyan Wu, Jingyi Sun, Hongyao Lou, Lu Xu, Chongyi Gan, Wenxin Liu, Yufeng Zhang, Rongqi Liang, Zhongfu Ni, Qixin Sun

**Affiliations:** State Key Laboratory of High-Efficiency Production of Wheat-Maize Double Cropping, Frontiers Science Center for Molecular Design Breeding (MOE), Key Laboratory of Crop Heterosis and Utilization (MOE), Beijing Key Laboratory of Crop Genetic Improvement, College of Agriculture and Biotechnology, China Agricultural University, Beijing, China

**Keywords:** combining ability, common wheat, genetic distance, heterosis, recurrent selection

## Abstract

Heterosis is an important approach to improving wheat yield and quality. Previously, five recurrent selection populations were established to broaden genetic diversity via a recurrent selection strategy using French wheat, Spelt wheat, spike-branched wheat, Tibetan semi-wild wheat and common wheat as distinct germplasm donors. The present study aimed to characterize and preliminary heterosis evaluate of these wheat recurrent selection populations after 20 cycles of recurrent selection, providing a reference for long-term recurrent selection to enhance inter-population genetic differentiation and heterosis potential. In this paper, 94 materials including recurrent selection lines and their donors, together with 120 common wheat cultivars/lines, were phenotyped for agronomic traits and genotyped with the wheat 90K SNP array to assess genotypic variation and genetic distance. Furthermore, 23 elite lines derived from different recurrent selection populations were used as parents in an incomplete NCII (inter-population crosses) and a full NCI(intra-population crosses) design to evaluate their genetic distance and the heterotic performance. The main results showed that significant phenotypic variation existed within each population, and its magnitude varied with traits and populations. Plant height (PH) and 1000-grain weight (TGW) showed evidence of convergent selection during population improvement, whereas spike length (SL) and mean grains per spike (GSN) displayed divergent selection, particularly within the four male parent populations (Set B). The SNP-based genetic distance and population differentiation patterns were basically consistent with the classification of exotic germplasm resources. The genetic differences of inter-populations were generally greater than those within PopulationI(Domestic wheat population). The general combining ability (GCA) differed across traits, while special combining ability (SCA) showed a significant correlation with parental phenotypic values. The proportion of crosses with positive mid-parent heterosis (MPH) ranged from 45.24% to 98.41%, varying with traits. Meanwhile, the percentage of crosses showing positive commercial heterosis (CH) was 58.73%–99.21%, which also differed across traits. Among all measured traits, TGW had the highest proportion of crosses with positive heterosis. MPH values within PopulationIwere less than that between PopulationI and other populations (Set B) in five traits (except GSN). Seven superior TGW crosses were identified, with CH exceeding 15% and specific combining ability (SCA) ranging from 1.56 to 13.06. A two-year field trial revealed that two of these crosses showed plot yield CH values of 4.81% and 8.54%, respectively. This study provides a reference for broadening genetic diversity for hybrid parents by recurrent selection using exotic germplasm resources, and preliminarily screened promising combinations, which provide an important reference for further investigations.

## 1 Introduction

Common wheat (Triticum aestivum L.) is a staple food crop for more than one-third of the world’s population (Shewry & Hey, 2015). With rapid economic development and continuous population growth, the global demand for wheat has been increasing steadily. Because arable land area cannot be expanded indefinitely, enhancing total wheat production relies heavily on increasing the yield per unit area. Heterosis utilization is one of the most effective strategies for improving wheat yield and quality (Longin et al., 2012; Jiang et al., 2017; Garst et al., 2023). Although wheat heterosis can increase grain yield by 3.5%–15%, hybrid wheat accounts for only 0.2% of the global wheat cultivation area (Singh et al., 2010; Revell et al., 2025; Gupta et al., 2019). Compared with hybrid rice and hybrid maize, the magnitude of heterosis in wheat is relatively low, which is the primary constraint limiting the large−scale application of hybrid wheat worldwide.

Due to the strict ecological adaptability of wheat, breeders usually select parental combinations from varieties within the same ecological region to develop hybrids with high heterosis. However, long−term directional genetic improvement has led to a narrow genetic base among modern wheat cultivars, characterized by close relatedness, limited genetic differences, and highly similar genetic backgrounds. This phenomenon directly results in low heterosis among elite cultivars and a shortage of strong heterotic combinations (Sun et al., 1996; Zhu et al., 2022; Boeven et al., 2020). For example, although more than 500 wheat varieties were released in China from the 1960s to the 2000s, but their pedigrees are largely derived from a limited number of backbone parents widely used both domestically and internationally (Zhuang, 2003; Zhou et al., 2007a, b; Cheng et al., 2020; Zhuang et al., 2024). Consequently, these varieties exhibit extremely high genetic similarity, leading to weak heterosis in intervarietal crosses.

To broaden the genetic diversity of hybrid wheat parents, our research center has established five recurrent selection populations using foreign common wheat, spelt wheat, ear-branched wheat, Tibetan semi-wild wheat, and Chinese common wheat as donor parents to respectively increase genetic divergence and introduce valuable variation (Sun et al., 1998; Du et al., 2002; Lu et al., 2007). The winter wheat RENAN released by INRA, France in 1989 (Dedryver et al., 2009) exhibits high yield, good quality and stripe rust resistance. Spelt lines are late-maturing, with plant heights exceeding 100 cm, long lax spikes and brittle rachises (Longin and Würschum, 2014; Rapp et al., 2016). Tibetan semi-wild wheat accessions Zang1817 and Zang1863 (Shao et al., 1980; Wang et al., 2004) showed extensive spike length variation, brittle rachises, and multiple florets/grains per spikelet. Spike-branched wheat Fen2 and Fen33 (Triticum aestivum L.), derived from common wheat ×Triticum turgidum crosses, possess multiple branched spikes and a higher grain number (Lu et al., 2007). These populations were constructed to develop new heterotic lines with distinct genetic backgrounds, utilizing the wheat dwarf male-sterile system (Liu and Yang, 1991). After more than ten cycles of recurrent selection, lines derived from the foreign wheat recurrent selection population exhibited significantly higher genetic diversity compared to conventional common wheat cultivars (Du et al., 2002). Using simple sequence repeat (SSR) markers, we further evaluated the genetic diversity of 45 wheat genotypes, including 20 common wheat accessions, 4 ear-branched wheat lines, 3 Tibetan wheat lines, 4 early spelt wheat mutants, and 14 lines derived from 15 cycles of recurrent selection. The results showed that the average genetic distances between common wheat and ear-branched wheat, Tibetan wheat, or Spelt wheat lines were significantly greater than those within the common wheat group (Lu et al., 2007). Therefore, with the continuous progression of recurrent selection, there remains a paucity of information regarding the rational selection of elite parental lines and the effective prediction of strong heterotic combinations, which hinders the further exploitation of heterosis in these novel populations.

Recurrent selection serves as a pivotal population improvement technique that enables the continuous pyramiding of favorable genes, expansion of genetic variation, and formation of genetically distinct heterotic groups, thereby alleviating the critical constraints of narrow genetic basis and limited heterosis potential in modern wheat breeding populations (Reif et al., 2005; Whitford et al., 2013). Although five genetically divergent wheat populations have been constructed and optimized via long-term recurrent selection in our previous research, the phenotypic variation characteristics, genome-wide genetic differentiation, combining ability, and heterosis potential of these populations remain unsystematically explored after multi-cycle artificial selection. Elucidating the phenotypic variation patterns and genetic differentiation profiles of these recurrent selection populations is essential to clarify the regulatory effects of long-term cyclic selection on population genetic improvement. Moreover, evaluating the combining ability and heterosis performance of intra- and inter-population hybrid combinations contributes to the screening of elite parental lines and superior hybrid crosses, which can provide valuable germplasm resources and theoretical references for breaking the bottleneck of insufficient heterosis in wheat. Notably, this study validates the efficacy of introducing exotic wheat germplasm to establish recurrent selection populations and heterotic groups, offering a feasible and sustainable strategy for the innovation of hybrid wheat parental materials, improvement of wheat heterosis utilization systems, and promotion of the large-scale popularization and application of hybrid wheat.

In this study, 94 materials including recurrent selection lines and their donor cultivars, together with 120 cultivars/lines, were analyzed for agronomic traits, genotypic variation, and genetic distance using the wheat 90K SNP array. Furthermore, 23 elite lines derived from different recurrent selection populations were used as parents in an incomplete NCII design to evaluate MPH and combining ability. This study aimed to explore whether long-term recurrent selection could enhance inter-population genetic differentiation and heterosis potential.

## 2 Materials and methods

### 2.1 Construction of the recurrent selection populations

The base population was established using the dwarf-sterile wheat (Liu and Yang, 1991), and then five recurrent selection populations were constructed respectively since 1991. High-yielding and stripe rust-resistant winter wheat cultivars/lines collected from the Northern China Winter Wheat Region, such as Jingdong 6, Nongda 3338, Jimai20 and other subsequent released cultivars, were used as donors for the Domestic Wheat Recurrent Selection Population (PopulationI) to breed high-yield lines with suitable agronomic traits for local production (**Supplementary Figure 1**).

Foreign common wheat, spelt wheat, ear-branched wheat, and Tibetan semi-wild wheat were used as donor parents for their unique agronomic traits to establish the Foreign Wheat Recurrent Selection Population (Population II), Spelt Recurrent Selection Population (Population III), Tibetan Semi-wild Wheat Recurrent Selection Population (Population IV), and Spike-branched Wheat Recurrent Selection Population (Population V), respectively.

**Figure 1:**
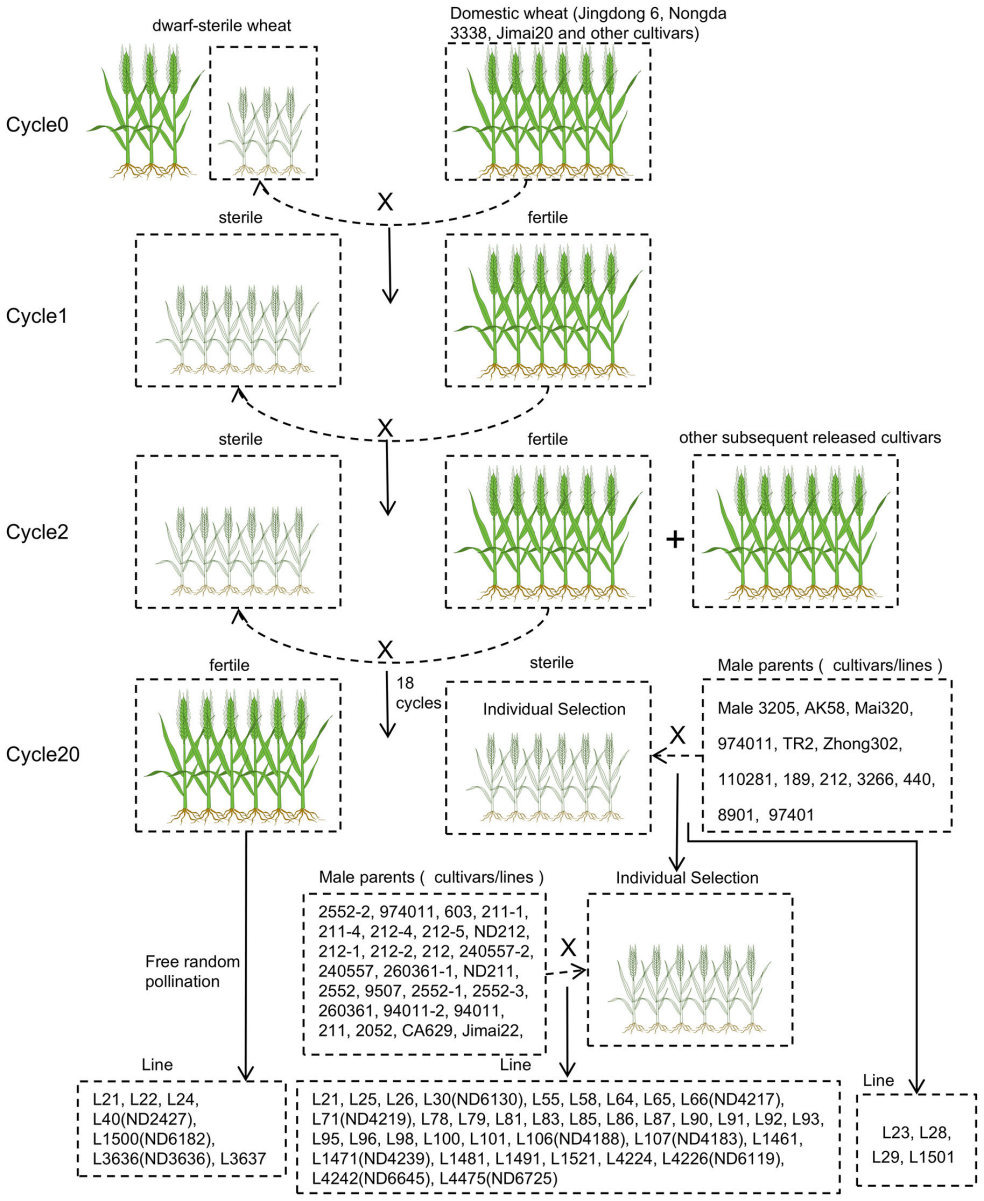
Pedigree Information of Lines from Domestic Wheat Recurrent Selection Populations.

Ideal individual plants (including fertile and male-sterile plants) were selected from the ongoing recurrent selection populations based on their distinct phenotypic performance, with a selection intensity of 5%–10%. Their progenies were bulked and randomly intercrossed (or backcrossed to donor plants) to develop an improved population for the next cycle of selection (**Table 1**, **Figure 1**, **Supplementary Figure 1**). Following 20 years of recurrent selection and several years of pedigree selection, many genetically stable lines with excellent comprehensive agronomic traits and abundant phenotypic diversity were developed, and most of them originated from two rounds of controlled crosses using male-sterile plants after the final cycle of recurrent selection. Some lines were further evaluated for heterotic performance (**Supplementary Table 1**, **Figure 1**, **Supplementary Figure 1**).

**Table 1:** Construction of five recurrent selection populations.

Populations	Initial donors	Selection criteria	Principal features
I	Domestic cultivars/lines	High yield, disease resistance	Suitable agronomic traits
II	Foreign “RENAN”	Heavy grain weight, alien blood	Alien blood
III	Spelt lines	Long spikes, heavy grain weight	Heavy weight per spike
IV	Tibetan semi-wild wheat	Multiple florets and grains	More grains per spike
V	Spike-branched wheat	Many spikelets and big grains	More and big grains

### 2.2 Plant materials and field experiments

Ninety-four recurrent selection lines and their donor materials, as well as 120 existing common wheat varieties (lines), were used for genetic diversity analysis, and heterotic groups were preliminarily classified via cluster analysis. Their specific names and categories are listed in **Supplementary Table 1**. This collection was planted at the Shangzhuang Experimental Station of China Agricultural University, Beijing in 2013–2014.

In this study, 10 elite lines with good comprehensive agronomic/yield traits and lower plant heights (70–75 cm) from PopulationI were designated as female parents (Set A). Concurrently, 13 elite lines characterized by high yield traits, unique traits and taller plant heights (75-85cm) from other populations were selected as male parents (Set B, **Table 2**). and thus 130 hybrid crosses were prepared according to the incomplete NCIImodel. Additionally, direct and reciprocal crosses among the 10 lines from Population I were performed using a Griffing I complete diallel cross design (Griffing, 1956). All crosses were obtained by artificial emasculation, bagging and pollination. The hybrid test collection, comprising the F1 seeds and their parents from both the NCII and GriffingIexperiments, was planted at the aforementioned station during the 2018–2019 season. Winter wheat cultivar Zhongmai 175 (ZM175), which served as the control in the official regional trials of the Northern Winter Wheat Region (2009–2019) and was bred by the Institute of Crop Science, Chinese Academy of Agricultural Sciences, was used as the control cultivar in this study. The selected crosses with strong heterosis in 1000-grain weight (TGW), together with ZM175, were subjected to yield plots trials during the 2021–2022 and 2022–2023 seasons.

**Table 2:** Elite parent lines selected for NCIIand griffing I combinations.

Lines	Populations	Set	Lines	Populations	Set
ND2427	I	A	F4113	II	B
ND3636	I	A	S4069	III	B
ND4183	I	A	S4070	III	B
ND4188	I	A	S5200	III	B
ND4219	I	A	S5363	III	B
ND4239	I	A	S5364	III	B
ND6119	I	A	X5345	IV	B
ND6130	I	A	X5353	IV	B
ND6645	I	A	Z4145	V	B
ND6725	I	A	Z4241	V	B
F4061	II	B	Z4316	V	B
F4071	II	B			

The plant materials were arranged in the field using a randomized complete block design with three replications. Each plot consisted of four rows with a 25-cm row spacing, 5-cm plant spacing, and 1.5-m row length. Each yield plot consisted of six rows, 3-m in length and 20-cm in row spacing, and was sown at a density of 3 million basic seedlings per hectare. Field management was performed according to local agronomic practices.

### 2.3 SNP genotyping and genetic distance analysis

Genomic DNA was extracted from young leaves following a standard CTAB method. Whole genome scanning of the samples was performed by Boao Jingdian Company using the Illumina Wheat 90K SNP array (Wang et al., 2014), and SNP genotyping was conducted by GenomeStudio Polyploid Clustering Module software v1.0 (Illumina Inc., San Diego, CA, USA).

The statistical software PowerMarker v3.25 was used to conduct genetic clustering analysis of the SNPs screened by polymorphism. Genetic distances between the wheat materials were calculated based on Nei’s (1972) genetic distance standard. The neighbor-joining (NJ) method was used to construct a phylogenetic tree (Saitou and Nei, 1987). Principal component analysis (PCA) and Kinship analysis were performed using Tassel v5.0, and the results were visualized in RStudio (2025.09.0 Build 387).

### 2.4 Phenotyping

At the mature stage, 10 representative plants were randomly selected from each plot to investigate their traits, including plant height (PH), spike length (SL), effective tiller number (ETN) and number of fertile spikelets per spike (FSN), and finally 10 plants were bulked and threshed to determine the average grain number per spike (GNS) and 1,000-grain weight (TGW) in the Seed Testing & Analysis Room.

### 2.5 Data analysis

The phenotype traits, combining ability and mid-parental heterosis were statistically analyzed using Microsoft Excel 2013, SPSS v23.0, and related software.

The heterosis of agronomic and yield traits was calculated using the following formulas: MPH= (F1 - MP)/MP × 100%, where MP = (P1 + P2)/2; CH = (F1 - CK)/CK × 100%, where CK is ZM175; High -parent heterosis (HPH) = (F1 - HP)/HP × 100%.

## 3 Results

### 3.1 phenotypic analysis of lines from different populations

Phenotypic analysis was conducted on six agronomic traits of the donor parents and their derived lines from different populations. The indicated showed that the values of six traits exhibited a normal or approximately normal distribution (**Table 3**, **Supplementary Figure 2**), which is characteristic of typical quantitative traits. Therefore, it is necessary to further improve the phenotypic performance of these populations.

**Table 3:** Statistical analysis of six traits of different populations.

Populations	PH/cm	SL/cm	ETN	FSN	TGW/g	GNS
I	75.73 ± 3.85	8.68 ± 0.74	12.04 ± 2.50	17.62 ± 1.55	40.29 ± 4.59	45.47 ± 9.56
II	83.97 ± 7.54	11.91 ± 1.84	8.72 ± 0.78	19.76 ± 1.95	40.70 ± 3.77	53.42 ± 4.45
III	74.18 ± 0.38	11.72 ± 1.78	13.72 ± 2.23	18.48 ± 0.63	40.74 ± 8.47	50.67 ± 7.68
IV	58.3 ± 3.82	11.67 ± 0.52	11.30 ± 0.14	16.6 ± 1.13	37.37 ± 0.44	64.62 ± 8.32
V	80.39 ± 3.85	9.04 ± 0.74	11.37 ± 2.50	18.49 ± 1.55	44.81 ± 4.59	46.24 ± 9.56
Ranges	47.4-95.4	6.2-14.9	6.0-18.8	14.2-23.6	23.6-59.5	34.9-80.2

Within a population, there were significant phenotypic variations among different lines, and the degree of variation were related to agronomic traits. For example, FSN showed the greatest variation in population V, whereas ETN exhibited the least variation in Population II. Across different populations, the characteristic traits of each population were generally consistent with the corresponding selection indices, reflecting a trend of selection-driven changes favorable to field production during recurrent selection and a pattern of divergent selection (**Table 3**, **Supplementary Figure 2**).

**Figure 2:**
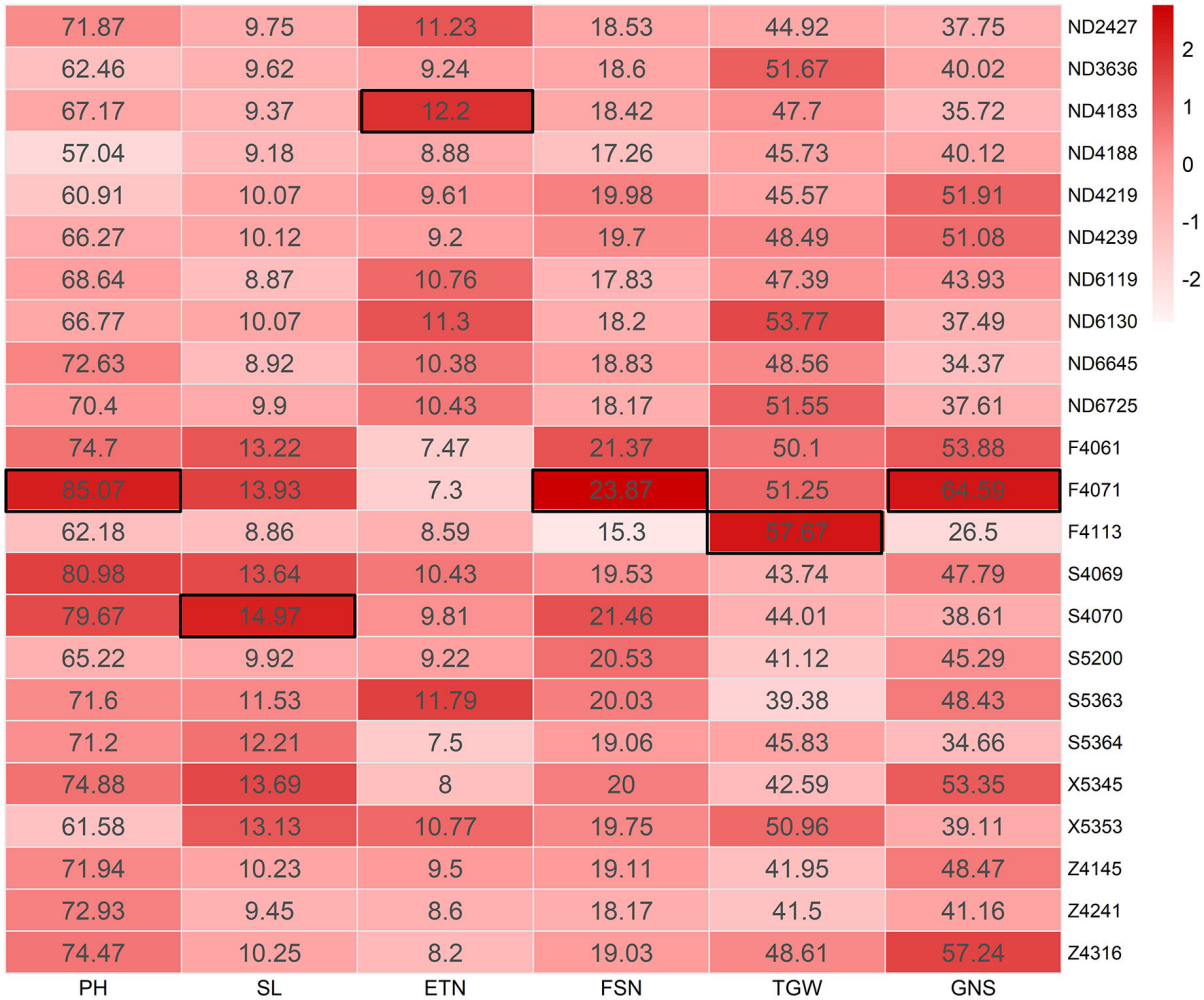
Heatmap of phenotypic values for 6 traits across 23 parent lines.

The elite parental lines selected for diallel cross combinations also exhibited a consistent trend with the aforementioned results (**Figure 2**). Specifically, the TGW of lines from Population II was higher than that of other groups, with the maximum of 57.67 g (F4113) and the minimum of 50.10 g (F4061). In contrast, the ETN of these lines was lower than that of the other populations. F4071 exhibited the optimal traits in PH, FSN, and GNS. The SL of lines from Population III was significantly longer than that of the other populations, with the maximum of 14.97 cm (S4070) and the minimum of 9.92 cm(S5200). X5345 and X5353 were characterized by early maturity, short PH, and low TGW. Three lines from Population V exhibited reduced SL, with PH ranging from 71.94 cm (Z4145) to 74.47 cm (Z4316).

Collectively, these results indicate that both the female parent population (Population I, Set A) and male parent populations (Populations II to V, Set B) exhibited convergent genetic improvement toward reduced PH and greater TGW, which were mostly associated with lodging resistance and high yield, reflecting the improvement of these traits and thus laying a robust experimental foundation for enhancing the over-standard heterosis in subsequent research.

### 3.2 Genotyping and Genetic distance analysis

In order to analyze the genetic diversity of these lines, their genotypes were profiled using the Illumina Wheat 90K SNP array. The genotyping analysis identified a total of 40,459 polymorphic SNPs, among which 27577 SNP loci exhibited a minor allele frequency (MAF) greater than 0.05. The number of polymorphic SNP loci varied substantially across chromosomes (**Supplementary Figure 3**), with the highest density observed on chromosome 2B (3,225) and the lowest polymorphic loci on chromosome 4D (751). This variation highlighted distinct chromosomal differences in genetic diversity.

Principal component analysis (PCA) indicates that all five recurrent selection progeny populations exhibit distinct clustering in the PCA plot. Accessions within the same population were concentrated with low dispersion, indicating small internal genetic differences. In contrast, different populations were clearly separated in the PCA space, reflecting significant genetic differentiation among them (**Supplementary Figure 4**). Overall, the genetic differences among populations were much greater than those within populations.

To compare the genetic variation and genetic differences among the materials from different populations, the genetic distances between these lines/cultivars were calculated according to SNP markers. The results indicate that the genetic distances ranged from 0 to 0.25 with the highest distribution density observed at 0.18 (**Table 4**). Within individual populations, Population I exhibited the largest average genetic distanc (0.165), followed by Population IV (0.160), Population III (0.130) and PopulationV(0.122), and PopulationII(0.110). Across different populations, the average genetic distance between PopulationVand III was the greatest (0.194), followed by PopulationVandII(0.190), PopulationVandIV(0.189), Population III andI(0.188) (**Table 4**). Cluster analysis and phylogenetic relationship analysis further confirmed these trends (**Supplementary Figure 5**, **Supplementary Figure 6**).

**Table 4:** Genetic distance analysis of SNP markers between different populations.

Populations	Population I	Population II	Population III	Population IV	Population V
Population I	0.165				
Population II	0.178	0.110			
Population III	0.188	0.180	0.130		
Population IV	0.181	0.179	0.156	0.160	
Population V	0.183	0.190	0.194	0.189	0.122

In general, the genetic differences of inter-Populations were generally greater than that intra-PopulationI, so these lines hold great potential to enrich the genetic base of hybrid wheat parental lines and widen the genetic distance between hybrid parents.

### 3.3 Combining ability analysis of elite lines from different populations

To select desirable parents and strongly heterotic combinations for future breeding, the combining ability of lines from different populations was analyzed using an incomplete North Carolina II (NC II) design. Variance analysis (ANOVA) of six traits showed that the variance between each cross reached a highly significant level, but the block variance was not significant, indicating that there were real genetic differences between the crosses (**Supplementary Table 3**).

Further variance analysis of combining ability (**Supplementary Table 3**) revealed that the general combining ability (GCA) variance for all six traits reached a highly significant level among the parents in Sets A and B. Additionally, the special combining ability (SCA) variance for most crosses reached a significant or highly significant level.

#### 3.3.1 Effect of the GCA

**Figure 3:**
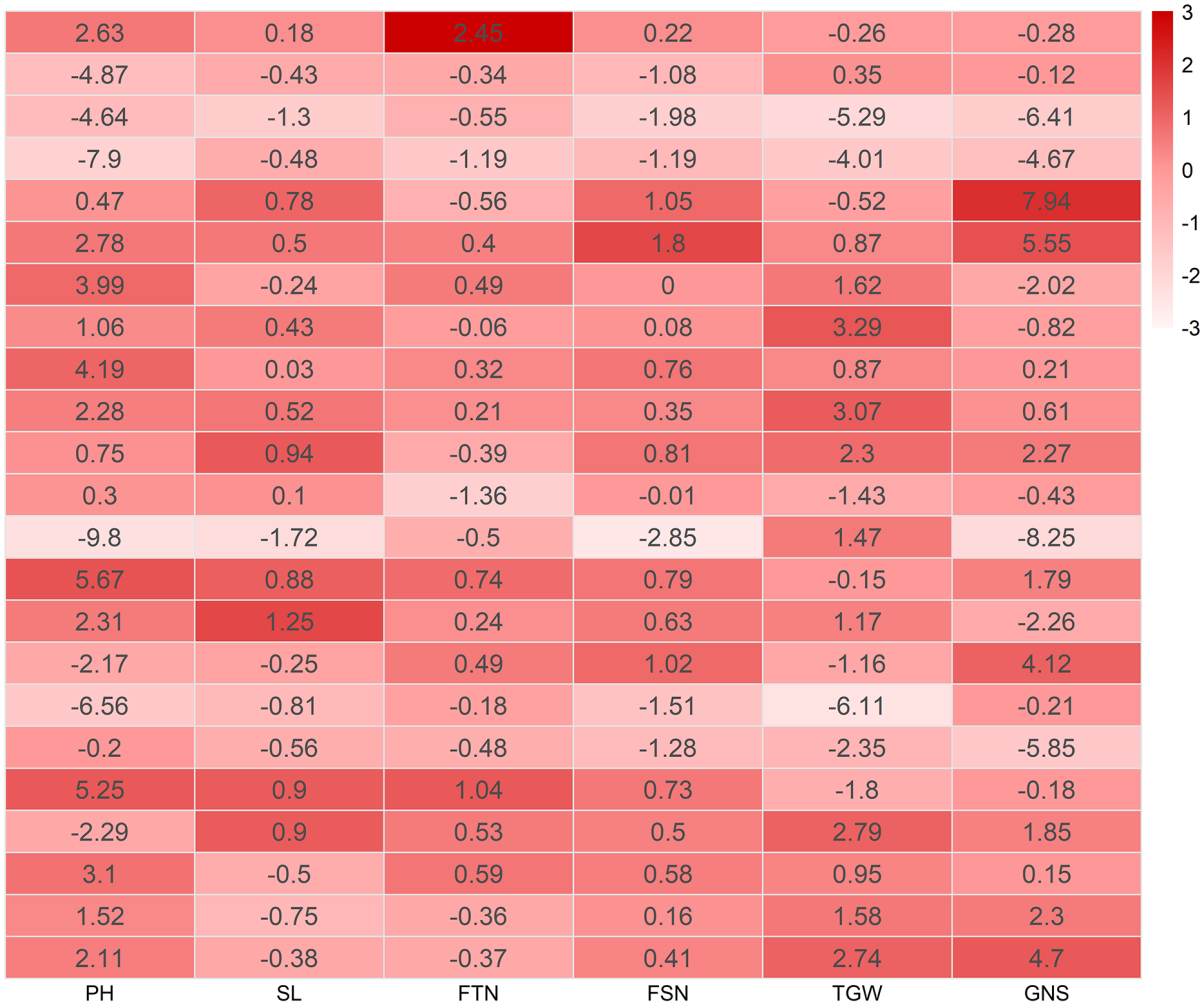
General combining ability (GCA) values of six traits of 23 elite parent lines.

The GCA values reflect the additive effect of genes and measure the ability of parents to transmit the values of traits to their offspring. To evaluate the 23 selected lines, the GCA values for the six traits were analyzed (**Figure 3**), The results indicate that the GCA values for a given trait vary substantially among different lines.

The GCA for all six traits of ND6725, ND6645 and ND4239 among the female parents (Set A, PopulationI) were all positive, indicating that these representative lines exhibit an increasing trend after recurrent selection. Notably, ND4239 had the highest GCA values for FSN and GNS among all parents. Conversely, lines S5364 and S5363 (Set B, Population III), along with ND4188 and ND4183 (Set A, PopulationI), exhibit the negative GCA values across all six traits, indicating that the performance of their F1 was likely to decline. The GCA for F4113 in PH, SL, FSN, and GNS was the lowest among all parents.

Parental phenotypic values were significantly correlated with their GCA values. Five traits displayed positive correlations at significant or highly significant levels (PH 0.60*, SL 0.62**, FSN 0.50*, TGW 0.48*, GNS 0.61**), whereas ETN was non-significantly and negatively correlated (-0.17). This suggested that parental selection based on superior target phenotypic performance was more desirable.

#### 3.3.2 The effect of the SCA

The SCA values reflect the non-additive effect of genes, and measure the gene interaction effect between two parents. To evaluate the SCA effects of the crosses, the SCA values for the six traits were analyzed (**Supplementary Table 4**, **Table 5**), The results indicate that the SCA effect for a given trait varies among different crosses (**Table 5**). Specifically, 69 crosses exhibited a positive SCA effect for PH, with a maximum of 22.21 in the cross ND4183/F4113 and a minimum value of -13.21 in ND4239/X5345.

**Table 5:** The SCA values of six traits of different crosses.

Traits	Positive crosses	Percentage	Maximum value	Minimum value
PH	69	54.76%	ND4183/F4113(22.21)	ND4239/X5345(-13.21)
SL	63	50.00%	ND4183/F4113(2.63)	ND4239/S4070(-1.55)
ETN	69	54.76%	ND4188/Z4145(3.72)	ND2427/Z2421(-2.94)
FSN	69	54.76%	ND4183/F4113(4.37)	ND4239/S4070 (-2.48)
TGW	68	53.97%	ND4183/F4113(13.06)	ND4239/X5345(-8.73)
GNS	70	55.56%	ND3636/Z4241(26.86)	ND4188/Z4241(-17.99)

The SCA values for different traits within the same cross can be either positive or negative. For example, the SCA values of ND6130/F4113 were 5.46 for TGW, and -1.06 for GNS. Furthermore, the SCA effect of a cross was not consistent with the GCA effects of its parents. For example, the SCA values of the cross ND4183/F4113 were positive and high for PH, SL, ETN, FSN and TGW, but their GCA values of these traits were negative.

#### 3.3.3 Genetic parameters

The genetic variance components and heritability for each trait were estimated using the random effect model (**Supplementary Table 5**).

The Vg values for PH, SL, FSN, and TGW were larger than the corresponding Vs, suggesting that these traits were mainly controlled by additive genetic effects. In contrast, the Vg values for ETN and GNS were close to their Vs values, indicating that these two traits were controlled by both additive and non-additive effects.

Heritability varies substantially among traits. SL and TGW showed high HB² and HN² values (greater than 50%), indicating strong genetic control and high efficiency for early-generation selection. GNS exhibited high HB² but low HN², implying weak heritability and suitability for delayed selection. PH, ETN, and FSN had low HB² and HN² (<50%), indicating strong environmental influence and suitability for selection in advance generations.

### 3.4 Heterosis analysis

To evaluate the recurrent selection populations and select elite heterotic crosses, the heterosis performance of the inter-population crosses between populationI and the other populations was analyzed (**Table 6**, **Supplementary Table S6**).

**Table 6:** The heterosis of six traits of all crosses.

Populations	MPH			HPH			CH		
Traits	Range	Mean	PCP	Range	Mean	PCP	Range	Mean	PCP
PH	-10.61-27.87	16.33	83.33	-17.10-18.87	-0.42	46.83	-15.74-32.78	10.65	88.89
SL	-14.60-14.10	1.18	60.32	-28.86-9.84	-8.74	16.67	-0.01-49.67	24.23	99.21
ETN	-27.43-44.43	-0.03	48.41	-35.81-34.88	-9.43	17.46	-34.09-49.21	3.08	55.56
FSN	-8.83-12.69	1.46	61.11	-17.44-9.56	-3.77	32.81	-8.08-26.16	10.34	95.24
TGW	-11.41-19.43	9.11	98.41	-16.52-16.05	3.30	78.57	-19.67-21.77	2.17	58.73
GSN	-30.42-59.60	0.32	45.24	-46.94-44.27	-10.43	24.60	-46.82-44.00	-5.72	36.51

Range, %, from the lowest heterosis to the highest heterosis. Mean, %, the average heterosis of all crosses. PCP, %, the percent of the crosses with positive heterosis in total crosses.

#### 3.4.1 MPH and PHP analysis

Most crosses exhibited the positive MPH for these traits, with TGW displaying the highest average MPH (9.11%), followed by PH (6.33%), FSN (1.46%), SL (1.18%), GNS (0.32%), and ETN (-0.03%).

The MPH for SL varied from -14.60% to 14.10%, and crosses with positive heterosis accounted for 60.32%, with the maximum value observed in the cross ND3636/Z4145(14.10%). The heterosis for FSN ranged from -8.83% to 12.69%, and 61.11% of the crosses showed a positive effect, with the strongest heterosis found in ND4239/F4113 (12.69%). Crosses with positive heterosis for GNS accounted for 45.24%, among which ND3636/Z4241 (59.60%) exhibited the highest value. The MPH for TGW ranged from -11.41% to 19.43%, and 98.41% of the crosses exhibited positive values, of which ND6119/Z4241 was the highest (19.43%).

Except for TGW, the HPH for the other five traits was negative, and their order was: TGW (3.30%) > PH (-0.42%) > FSN (-3.77%) > SL (-8.74%) > ETN (-9.43%) > GSN (-10.43%). The HPH for TGW ranged from -16.52% to 16.05%, with 78.57% of the crosses showing positive heterosis, and ND6119/Z4316 (16.05%) performed the best. For GNS, the HPH varied from -46.94% to 44.27%, with 24.60% of the crosses showing positive heterosis, and the best cross was ND3636/Z4241 (44.27%).

#### 3.4.2 CH analysis

The potential for widespread promotion of a hybrid wheat cultivar in production depends on its yield heterosis exceeding that of the dominant commercial cultivars. The average CH for the five traits was positive, with SL displaying the highest average value (24.23%), followed by PH (10.65%), FSN (10.34%), ETN (3.08%), TGW (2.17%) and GNS (-5.72%). The CH for SL ranged from -0.01% to 49.67%, and 99.21% of the crosses exhibited positive heterosis, with ND4219/S4070 showing the strongest performance (49.67%). The CH for TGW varied from -19.67% to 21.77%, and 58.73% of the crosses were positive, of which the most prominent cross was ND6130/F4113 (21.77%).

Taking TGW as the representative trait, further analysis of its CH heterosis (**Table 7**) showed that 7 crosses exhibited 15% CH. F4113 was the male parent in four crosses, while ND4183, ND6130, and ND6725 each served as the female parent in two crosses. These 7 crosses demonstrated superior performance: their MPH ranged from 6.50% to 19.04%, CH from 1.02% to 12.34%, and SCA effects from 1.56% to 13.06, while their corresponding parents generally possessed high GCA values. Notably, ND418/F4113 exhibited a high SCA effect but low GCA values in both parents yet it still exhibited strong heterosis, which could be attributed to the complementary yield components of the two parents based on their phenotypes.

**Table 7:** Comprehensive evaluation of the strong heterotic crosses in TGW.

Crosses	GCA/♀	GCA/♂	SCA	MPH	CH
ND6130/F4113	3.29	1.47	5.46	8.14%	21.77%
ND4183/F4113	-5.29	1.47	13.06	12.53%	19.04%
ND6725/S5364	3.07	-2.35	7.19	19.04%	17.51%
ND6725/F4113	3.07	1.47	5.97	10.92%	16.83%
ND6130/X5353	3.29	2.79	1.56	10.10%	16.71%
ND4183/F4061	-5.29	2.3	8.79	14.12%	16.12%
ND3636/F4113	0.35	1.47	6.38	6.50%	15.03%

**Figure 4:**
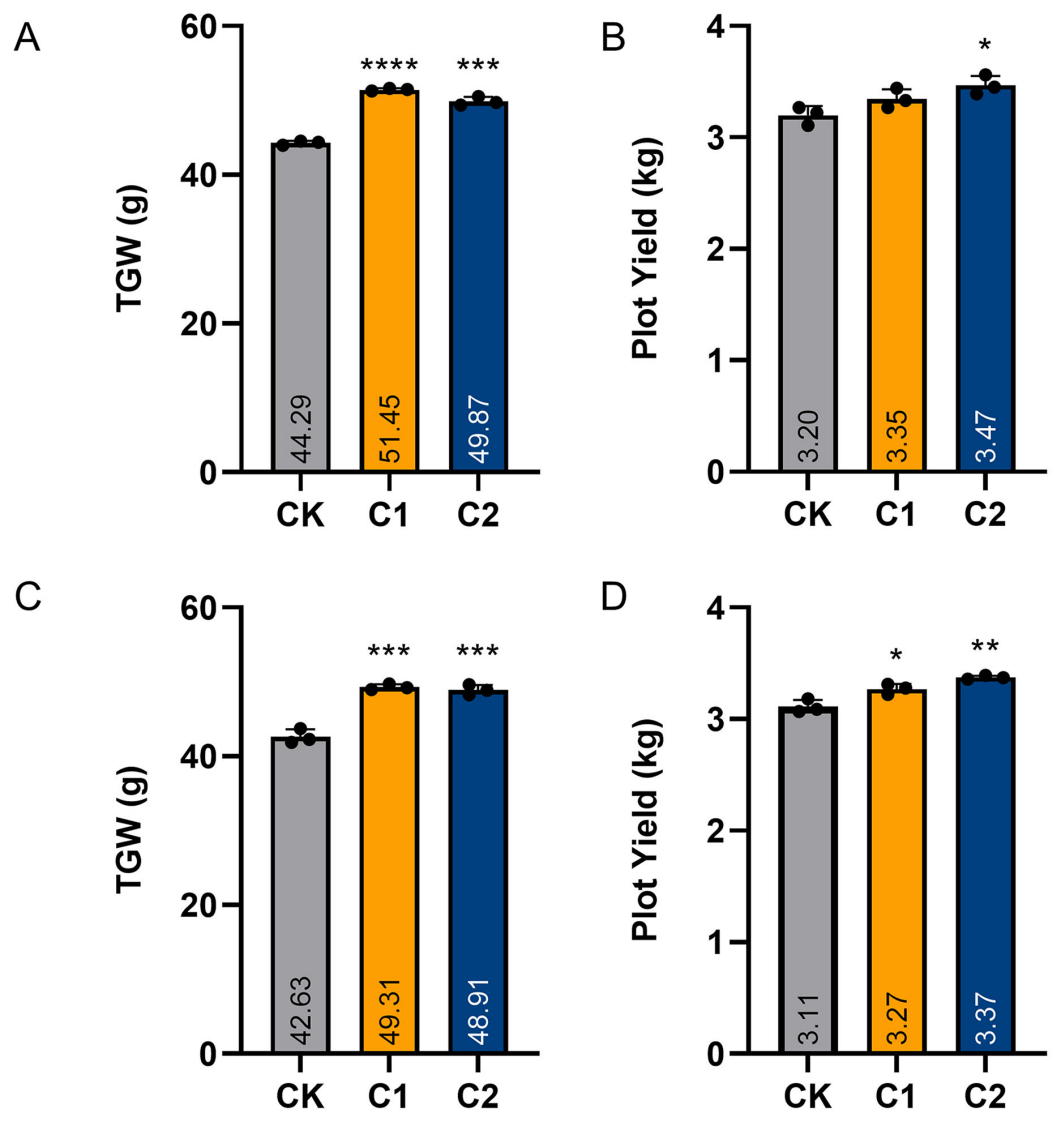
Yield evaluation of ND6130/F4113 (abbreviated as C1) and ND6725/S5364 (abbreviated as C2) in 2022 **(A, B)** and 2023 **(C, D)**. *: significance difference at 5%, **: significance difference at 1%, ***: significance difference at 0.1%, ****: significance difference at 0.01%.

The crosses ND6130/F4113 (abbreviated as C1) and ND6725/S5364 (abbreviated as C2) were selected for further yield evaluation (**Figure 4**). The two-year regional trial results showed that under production conditions, C1 exhibited CH for TGW of 16.17% and 15.67%, respectively, while C2 exhibited 12.60% and 14.74%; for plot yield, C1 showed 4.58% and 5.03% CH, respectively, and C2 8.33% and 8.74%.

#### 3.4.3 Relationship analysis between combining ability and heterosis

Correlation analysis between SCA and heterosis (**Supplementary Table 9**) showed the SCA was significantly positively correlated with MPH, and closely correlated with HPH and CH in 5 traits except FSN. In addition, GCA had no or negative correlation with MPH/HPH.

### 3.5 Heterosis analysis of intra- and inter-populations

To compare the heterotic performance of crosses from different populations, the heterosis of intra-population I was further analyzed (**Table 8**, **Supplementary Table S6**). The results indicate that TGW exhibited the highest average MPH (6.63%), followed by GNS (6.01%), SL (2.18%), PH (2.11%), FSN (0.83%), and ETN (-5.00%). Among these traits, 91.01% of the crosses exhibited positive heterosis for TGW. Meanwhile, both ND4188/ND4183 and ND4183/ND4188 showed high MPH for TGW.

**Table 8:** MPH of intra- and inter-population crosses.

Crosses	PH	SL	ETN	FSN	GNS	TGW
PI×PII	4.62%	2.29%	1.33%	0.39%	-0.78%	7.30%
PI×III	6.52%	0.61%	-0.78%	1.13%	2.95%	9.79%
PI×IV	8.64%	-0.35%	3.95%	1.36%	3.24%	5.98%
PI×PV	6.09%	2.07%	-2.77%	3.04%	1.04%	11.78%
PI×PI	2.13%	2.15%	-4.86%	0.87%	0.14%	6.64%

For a certain trait, the heterosis varied among the crosses between different populations (**Table 8**, **Supplementary Table S7**). The crosses between PopulationIand Population II lines had the high MPH in SL. The crosses between PopulationIand Population III lines had the high MPH in GSN. The crosses between PopulationIand Population IV lines had the high MPH in PH and ETN. The crosses between PopulationIand Population Vhad the high MPH in SL, FSN, and TGW. There was no significant linear correlation between inter-population heterosis and inter-population genetic distances, though a certain trend was observed.

A comparison of heterosis within and between populations revealed that the MPH within PopulationI(Set A) was lower than those between PopulationI(Set A) and other populations (Set B) for five of the traits (except GNS). This result was consistent with the above result that genetic differences between populations were generally greater than those within Population I, indicating that enriching the genetic basis of parents could improve the heterosis of hybrid wheat by crossing Set B with Set A.

## 4 Discussion

### 4.1 The phenotypes and genetic distances of wheat genetically divergent populations

The core of recurrent selection lies in improving progeny lines through population improvement methods. In this process, it is necessary to continuously select and accumulate elite populations formed by differentiation, while selecting select lines within the population through testcrossing. Pedigree analysis combined with phenotypic classification represents a conventional and fundamental strategy, which is easy to implement but restricted by relatively low resolution and accuracy. Molecular marker-based clustering, dominated by SSR and SNP markers, offers an objective and efficient tool for categorizing genetic groups with high repeatability (Zhou et al., 2018).

In previous studies on wheat ecological regions, the main agronomic and yield traits of parents varied regionally: 1) Huanghuai Winter Wheat Region: PH 75–85 cm, SL 7.5–9.0 cm, spikes per plant 4.5–6.0, GNS 32–38, TGW 42–47 g, and grain yield per plant 18–24 g (Shi et al., 2013; Xue et al., 2025); 2) Northern Winter Wheat Region: PH 72–82 cm, GNS 30–36, TGW 40–45 g, grain yield per plant 16–22 g; 3)The Yangtze River Wheat Region: PH 78–88 cm, GSN 34–40, TGW 41–46 g (Li et al., 2021). In the present study, the values of PH and TGW were similar to those in the Northern Winter Wheat Region, indicating a pattern of convergent selection during population improvement, as lower PH and higher TGW contribute to lodging resistance and yield. By contrast, SL and GNS exhibited divergent selection across the four male parent populations (Set B), indicating that these exotic resources could improve the population potential for heterosis.

In our study, the genetically divergent populations were initially initial divided according to their germplasm sources. Based on 32 pairs of random amplified polymorphic DNA (RAPD) primers, Tibetan semi-wild wheat (7 accessions) and common wheat were clearly separated into two groups, with mean genetic distances of 0.4319 within Tibetan semi-wild wheat and 0.2164 within winter common wheat. Therefore, Tibetan semi-wild wheat could be used to broaden the genetic basis for hybrid wheat breeding (Ni et al., 1997). Lu et al. used 23 pairs of SSR marker to evaluate the genetic diversity of 45 selected wheat genotypes, and the SSR-based genetic distance between common wheat varieties ranged from 0.404 to 0.662, whereas wider genetic differentiation was detected between common wheat and spelt wheat (Population III), Tibetan semi-wild wheat (PopulationIV), and branch-spike wheat (PopulationV), as well as populations improved after more than 10 rounds of recurrent selection, with values reaching 0.9217, 0.9087, 0.8145, and 0.7715, respectively (Liu et al., 2007). In the present study, significant differences were observed in the count of polymorphic SNP loci across chromosomes (**Supplementary Figure 3**), and chromosome 2B had richer genetic diversity while chromosome 4D showed more conserved genetic traits. This variation might be primarily associated with chromosome size, recombination activity, and selection pressures, as well as the design of the Illumina 90K SNP chip, thereby affecting genetic distance values. The SNP-based average genetic distance of populations ranged from 0.122 to 0.165, and the average genetic distance between PopulationVand III was biggest (0.194), followed by PopulationV&II(0.190), PopulationV&IV(0.189), Population III &I(0.188) after more than 20 rounds of recurrent selection. Differences existed in inter- and intra-population genetic distances revealed by the three types of molecular markers. In addition to the populations being from different recurrent selection stages, genome-wide SNP markers are superior to RAPD and SSR in assessing genetic distance, owing to the limited quantity of the latter two markers. However, the significant correlation between genetic distance and heterosis was absent in this study.

Collectively, our results demonstrate that genetic distance, phenotypic traits, and exotic germplasm resources were descriptive metrics for evaluating genetically divergent populations.

### 4.2 The combining ability and heterotic performance of genetically divergent groups

Analysis of heterosis among different domestic wheat producing regions showed that GCA contributed 40%–50% to the genetic variation of wheat grain yield, while SCA accounted for 50%–60% (Shi et al., 2013; Adhikari et al., 2020). The probability of obtaining elite heterotic crosses exceeded 75% when the total GCA of both parents was greater than +3.0, the SCA of the hybrid cross is higher than +2.0, indicating that genetically divergent heterotic groups contribute greatly to strong yield heterosis in wheat (Chen et al., 2022; Saavedra-vila et al., 2025). In this paper, parental phenotypic values were significantly correlated with GCA, and five traits (with the exception of ETN) displayed positive correlations at significant or highly significant levels, ranging from 0.48 to 0.62. Therefore, these correlations can serve as a reference for the parental assignment between different groups in wheat hybrid breeding programs.

In terms of intra-population heterotic performance, the inter-varietal recurrent selection population was dominated by common wheat varieties/lines, and the MPH for the yield per plant was only 11.14%, showing stable but limited heterosis, thus belonging to the basic application heterotic group. Strong heterotic crosses exhibited MPH of 12.0%–25.0%, HPH of 8.0%–18.0%, and CH of 2.00%–10.03% for grain yield. Meanwhile, CH heterosis for GNS and TGW varied from 4.22% to 30.54% and 1.32% to 12.92%, respectively (Shi et al., 2013);. In this study, TGW exhibited the highest average MPH at 6.63%, followed by GNS (6.01%), SL (2.18%), PH (2.11%), FSN (0.83%), and ETN (−5.00%). These results further indicate that intra−population MPH among domestic wheat materials was relatively limited.

Interspecific lines could significantly increase their genetic differences and heterosis magnitude. We previously reported that the average heterosis in the yield per plant was 69.4% using common wheat materials and Spelt (Sun et al., 1998), and even up to 109.2% (43.14%-187.6%); meanwhile MPH of TGW ranged from -4.24% to 58.73%, with an average of 28.43% (Dou et al., 2002). MPH of the yield per plant in interspecific crosses between common wheat and spelt reached 111.39%, which was considerably higher than the 11.14% observed in intraspecific crosses among common wheat varieties, and a significantly positive correlation was detected between genetic distance and heterosis strength (Cui et al., 2002). Between common wheat and compactum wheat, the mid-parent heterosis for yield can reach 77.19%, and the heterosis of yield components is extremely significantly positively correlated with yield level (Yang et al., 2004). In the aforementioned studies, interspecific hybrid combinations between common wheat and distant relatives such as spelt wheat and compactum wheat exhibited an ultra-high heterosis of 70%–111.39%. However, such heterosis was obtained under non-adaptive environments and thus is not feasible for agricultural production.

In this paper, the lines from population III overcame the shortcomings of spelt by recurrent selection, and enriched their genetic base as female parents, the average MPH for TGW was 9.79% with the range of 2.21%-19.04%, while the average CH was -1.06% compared with the commercial cultivar ZM175. Because the MPH of TGW was obtained by comparing the crosses with highly the improved elite parental lines, the heterosis values are clearly lower than that of original unimproved germplasm. Meanwhile, CH of yield was compared with the widely cultivated ZM175, which possesses strong tillering and spike-forming abilities, and it is difficult to achieve a yield advantage of more than 5%. However, the preliminary heterosis evaluation and yield plot trials in this study were conducted only at a single experimental site. This research has certain limitations and deficiencies, including a limited number of environmental conditions, the absence of validation populations, and the low predictive efficiency of genetic distance, which need to be further investigated in future studies.

Hybrid wheat production relies mainly on yield heterosis rather than TGW. However, all F_1_ seeds in this study were obtained through manual emasculation and pollination, and thus limited hybrid seeds were available for plot yield evaluation during the heterosis analysis. Therefore, we first selected TGW (one of the three major yield components) as the representative trait for further CH heterosis analysis to preliminarily evaluate and screen cross combinations. Promising crosses will be further evaluated for plot yield in subsequent multi-environment trials.

Conclusively, by introgressing favorable genes/traits of alien germplasm into common wheat via recurrent selection, the MPH of crosses between recurrently selected common wheat lines and alien lines was reduced. Nevertheless, the agronomic and yield traits of these lines have become comparable to commercial cultivars, enabling preliminary feasibility for hybrid production.

## Data Availability

The datasets presented in this study can be found in online repositories. The names of the repository/repositories and accession number(s) can be found in the article/**Supplementary Material**.

## Supplementary material

Supplementary Figure 1 Pedigree Information of Lines from Foreign Wheat, Spelt, Tibetan semi-wild wheat, and Spike-branched wheat Recurrent Selection Populations.

Supplementary Figure 2 Phenotypic Distribution of Six Traits in Populations Derived from Different Cycles of Recurrent Selection.

Supplementary Figure 3 Distribution of Markers on Each Chromosome.

Supplementary Figure 4 (PCA) of 214 Wheat Lines/Cultivars.

Supplementary Figure 5 Clustering Analysis of 214 Wheat Lines/Cultivars.

Supplementary Figure 6 Kinship Matrix of 214 Wheat Lines/Cultivars.

Supplementary Table 1 List of 214 wheat lines/cultivars used for SNP Genotyping.

Supplementary Table 2 Analysis of Variance for Blocks and different combinations.

Supplementary Table 3 Analysis of combining ability variance for six traits of all tested lines.

Supplementary Table 4 SCA effects of six traits in all NC-II crosses.

Supplementary Table 5 Estimation of genetic parameters for six traits.

Supplementary Table 6 The Mid-parent and over-high parent heterosis (MPH and PHP) of six traits in NC II crosses (intra-populations).

Supplementary Table 7 The MPH of crosses from different lines within Population 1.

Supplementary Table 8 The CH of six traits in NC II crosses.

Supplementary Table 9 The relationship between SCA and heterosis.

## References

[B500] Adhikari A. Ibrahim A. M. H. Rudd J. C. Baenziger P. S. Jean-Benoit (2020). Supplementing selection decisions in a hybrid wheat breeding program by using F2 yield as a proxy of F1 performance. Euphytica 216(8), 126. doi: 10.1007/s10681-020-02664-0 30311153

[B501] Boeven P. H. G. Zhao Y. Thorwarth P. Liu F. Maurer H. P. Gils M. (2020). Negative dominance and dominance-by-dominance epistatic effects reduce grain-yield heterosis in wide crosses in wheat. Sci. Adv. 6(24), eaay4897. doi: 10.1126/sciadv.aay4897 32582844 PMC7292627

[B502] Chen X. D. Wu X. J. Fang F. Song L. T. Dong N. Hu T. Z. (2022). Prediction of wheat heterosis based on SNP genetic distance and combining ability. Chin. Sci. Bull. 67(26), 3221–3232. doi: 10.1360/TB-2022-0358

[B2] Cheng Y. Zhang J. Wu B. Wang L. He Z. H. (2020). Resequencing of 145 landmark cultivars reveals asymmetric sub-genome selection and strong founder genotype effects on wheat breeding in China. Mol. Plant 13, 1895–1908. doi: 10.1016/j.molp.2020.09.001 32896642

[B503] Cui G. H. Ni Z. F. Wu L. M. Li Y. Q. Sun Q. X. (2002). Study on wheat heterotic group V. Relationships between microsatellite markers-based genetic distance and heterosis between *T. aestivum* and *T. spelta*. J. Triticeae Crops 22(1), 10–14. doi: 10.3969/j.issn.1009-1041.2002.01.002

[B3] Dedryver F. Paillard S. Mallard S. Robert O. Trottet M. Nègre S. (2009). Characterization of genetic components involved in durable resistance to stripe rust in the bread wheat 'Renan'. Phytopathology 99, 968–973. doi: 10.1094/phyto-99-8-0968 19594316

[B4] Dou B. D. Sun Q. X. Ni Z. F. Wu L. M. Meng F. R. Liu B. S. (2002). Study on heterosis of interspecific hybrid wheat: I. The yield and quality heterosis of interspecific hybrid between common wheat, spelt wheat and club wheat. J. China Agric. Univ. 7 (1), 47–53. doi: 10.3321/j.issn:1007-4333.2002.01.010 30704229

[B5] Du J. K. Yao Y. Y. Ni Z. F. Peng H. R. Sun Q. X. (2002). Genetic Diversity Revealed by ISSR Molecular Marker in Common Wheat, Spelt, Compactum and Progeny of Recurrent Selection. Acta Genet. Sin. 29 (5), 445–452. doi: CNKI:SUN:YCXB.0.2002-05-012 12043574

[B504] Garst N. Belamkar V. Easterly A. Guttieri M. J. Stoll H. Ibrahim A. M. H. (2023). Evaluation of pollination traits important for hybrid wheat development in Great Plains germplasm. Crop Sci. 63, 1169–1182. doi: 10.1002/csc2.20926 41531421

[B6] Gilbert N. McVetty P. B. E. Rimmer S. R. (2000). Hybrid wheat: history, current status, and future prospects. Plant Breed. Rev. 19, 137–182.

[B7] Griffing B. (1956). Concept of general and specific combining ability in relation to diallel crossing systems. Aust. J. Biol. Sci. 9, 463–493. doi: 10.1071/bi9560463 38477348

[B505] Gupta P. K. Balyan H. S. Gahlaut V. Saripalli G. Joshi A. K. (2019). Hybrid wheat: past, present and future. Theor. Appl. Genet. 132(4), 823–843. doi: 10.1007/s00122-019-03397-y 31321476

[B506] Jiang Y. Schmidt R. Zhao Y. Reif J. C. (2017). A quantitative genetic framework highlights the role of epistatic effects for grain-yield heterosis in bread wheat. Nat. Genet. 49, 1741–1746. doi: 10.1038/ng.3974 29038596

[B10] Li N. N. Du X. Y. Han Y. L. Zou S. K. Li S. C. Wang L. N. (2021). Comprehensive Analysis of Agronomic Traits of Four Main Winter Wheat Producing Areas in China. Seed 40 (12), 94–101. doi: 10.16590/j.cnki.1001-4705.2021.12.094

[B12] Liu B. Yang L. (1991). Breeding of dwarfing-sterile wheat and its potential values in wheat breeding. Chin. Sci. Bull. 36(4), 306–308.

[B13] Liu J. Liu L. Hou N. Zhang A. Liu C. (2007). Genetic diversity of wheat gene pool of recurrent selection assessed by microsatellite markers and morphological traits. Euphytica 155 (1-2), 249–258. doi: 10.1007/s10681-006-9326-x 30311153

[B14] Longin C. Mühleisen J. Maurer H. P. Zhang H. Gowda M. Reif J. C. (2012). Hybrid breeding in autogamous cereals. Theor. Appl. Genet. 125, 1087–1096. doi: 10.1007/s00122-012-1967-7 22918662

[B16] Longin C. F. H. Ziegler J. Schweiggert R. Koehler P. Carle R. Würschum T. (2016). Comparative Study of Hulled (Einkorn, Emmer, and Spelt) and Naked Wheats (Durum and Bread Wheat): Agronomic Performance and Quality Traits. Crop Sci. 56, 302–311. doi: 10.2135/cropsci2015.04.0242

[B17] Longin C. F. H. Würschum T. (2014). Genetic variability, heritability and correlation among agronomic and disease resistance traits in a diversity panel and elite breeding material of spelt wheat. Plant Breed. 133, 459–464. doi: 10.1111/pbr.12182 40046247

[B18] Lu L. H. Li Z. X. Ni Z. F. Peng H. R. Nie X. L. Sun Q. X. (2007). Study on wheat heterotic group VI. Genetic diversity revealed by SSR marker between common wheat, ear-branched wheat, wheat lines derived from recurrent selection, Tibetan wheat and early spelt wheat mutant lines. J. Triticeae Crops 27(2), 201–206. doi: CNKI:SUN:MLZW.0.2007-02-005

[B1000] Nei M. (1972). Genetic distance between populations. Am. Nat. 106 (949), 283–292. doi: 10.1086/282771 24979626

[B21] Ni Z. F. Sun Q. X. Liu Z. Y. Huang T. C. (1997). Study on wheat heterotic group II. Genetic diversity revealed by RAPD in *Triticum aestivum*, *Triticum aestivum* ssp. *tibetanum* and *Triticum spelta*. J. Agric. Biotechnol. 5(2), 103–111. doi: CNKI:SUN:NYSB.0.1997-02-000

[B22] Rapp M. Longin C. F. H. Würschum T. (2016). Phenotypic characterization of a worldwide spelt wheat collection. Crop Sci. 56, 2939–2948.

[B23] Reif J. C. Hallauer A. R. Melchinger A. E. (2005). Heterosis and heterotic patterns in maize. Maydica 50, 215–223.

[B507] Revell I. Zhang P. Dong C. Salter W. T. Trethowan R. (2025). Heterosis in wheat: mechanisms, benefits, and challenges in hybrid development. J. Exp. Bot. doi: 10.1093/jxb/eraf159 40231743 PMC13139662

[B508] Saavedra-Vila J. I. Gerard G. S. Esposito S. Govindan V. Huerta-Espino J. Tadesse Z. (2025). Unraveling the genetic basis of general combining ability in CIMMYT elite bread wheat germplasm: implications for breeding strategies optimization. Front. Plant Sci. 16, 1675993. doi: 10.3389/fpls.2025.1675993 41180394 PMC12575131

[B24] Saitou N. Nei M. (1987). The neighbor-joining method: a new method for reconstructing phylogenetic trees. Mol. Biol. Evol. 4(4), 406–425. doi: 10.1006/rwgn.2001.1479 3447015

[B25] Shao Q. Li C. Basangciren (1980). Semi-wild wheat from Xizang (Tibet). Acta Genet. Sin. 7(2), 149–156.

[B27] Shewry P. R. Hey S. J. (2015). The contribution of wheat to human diet and health. Food Energy Secur. 4, 178–202. doi: 10.1002/fes3.64 27610232 PMC4998136

[B509] Shi X. X. Bi X. J. Ma S. C. Qi J. J. Han F. F. Zhang G. S. (2013). Heterosis and combining ability analysis of parents for hybrid wheat in Huanghuai wheat region. J. Triticeae Crops 33(6), 1110–1118.

[B510] Singh S. Chatrath R. Mishra B. (2010). Perspective of hybrid wheat research: a review. Indian J. Agric. Sci. 80(12), 1013–1027.

[B29] Sun Q. X. Huang T. C. Ni Z. F. Procunier J. D. (1996). Study on wheat heterotic group I. Genetic diversity among wheat varieties revealed by RAPD markers. J. Agric. Biotechnol. 4 (2), 103–110.

[B31] Sun Q. X. Ni Z. F. Liu Z. Y. Chen X. Y. Gao J. H. (1998). Heterosis of inter-specific hybrids between common wheat and spelt wheat. J. China Agric. Univ. 3 (1), 10. doi: CNKI:SUN:NYDX.0.1998-01-003

[B1100] Wang S. Wong D. Forrest K. Allen A. Chao S. Huang B. E. . (2014). Characterization of polyploid wheat genomic diversity using a high-density 90,000 single nucleotide polymorphism array. Plant Biotechnol. J. 12 (6), 787–796. doi: 10.1111/pbi.12183 24646323 PMC4265271

[B33] Wang Z. Q. Zheng Y. L. (2004). Advances on the study of Tibetan semi-wild wheat. J. Triticeae Crops. 4, 133–35.

[B35] Whitford R. Fleury D. Reif J. C. Garcia M. Okada T. Langridge P. (2013). Hybrid breeding in wheat: Technologies to improve hybrid wheat seed production. J. Exp. Bot. 64, 5411–5428. doi: 10.1093/jxb/ert333 24179097

[B1200] Xue Z. W. Song H. Zhang Y. F. Yang C. L. (2025). Comprehensive analysis of agronomic and quality traits of new wheat varieties (lines) from different ecological regions. Jiangsu Agric. Sci. 53 (16), 99–111.

[B1300] Yang X. Q. Liu P. Han Z. F. Ni Z. F. Sun Q. X. (2004). Study on genetic differences of SSR and EST-SSR molecular markers in genomes of common wheat, spelt wheat and club wheat. Prog. Nat. Sci. 14 (9), 10–10. doi: 10.3321/j.issn:1002-008X.2004.09.005 30704229

[B1400] Zhou Z. Zhang C. Lu X. Wang L. Hao Z. Li M. . (2018). Dissecting the Genetic Basis Underlying Combining Ability of Plant Height Related Traits in Maize. Front. Plant Sci. 9, 1117.doi: 10.3389/fpls.2018.01117 30116252 PMC6083371

[B1500] Zhou Y. He Z. H. Sui X. X. Xia X. C. Zhang X. K. Zhang G. S. (2007a). Genetic improvement of wheat yield potential in north China. Springer Netherlands. doi: 10.1007/1-4020-5497-1_70

[B1600] Zhou Y. He Z. H. Sui X. X. Xia X. C. Zhang X. K. Zhang G. S. (2007b). Genetic Improvement of Grain Yield and Associated Traits in the Northern China Winter Wheat Region from 1960 to 2000. Crop Sci. 47, 245–253. doi: 10.2135/cropsci2006.03.0175

[B1700] Zhu X. Xu Y. Li J. Zhang X. (2022). Establishment of heterotic groups for hybrid wheat breeding. Chin. Sci. Bull. 67 (26), 3152–3164. doi: 10.1360/tb-2022-0392

[B37] Zhuang L. Liu H. Hou J. Wang D. Zhang L. Wang Y. (2024). Genetic improvement of important agronomic traits in Chinese wheat breeding over the past 70 years. BMC Plant Biol. 24, 1151. doi: 10.1186/s12870-024-05841-8 39614144 PMC11606123

[B38] Zhuang Q. S. (2003). Chinese wheat varieties and their pedigrees (Beijing: China Agriculture Press), 1–586.

