# Supplementary figures and images for: Characterization and preliminary heterosis evaluation of novel wheat genetically divergent populations
Source: Front Plant Sci. 2026 Jun 22;17:1824151. doi: 10.3389/fpls.2026.1824151 (PMC13333715; doi:10.3389/fpls.2026.1824151)

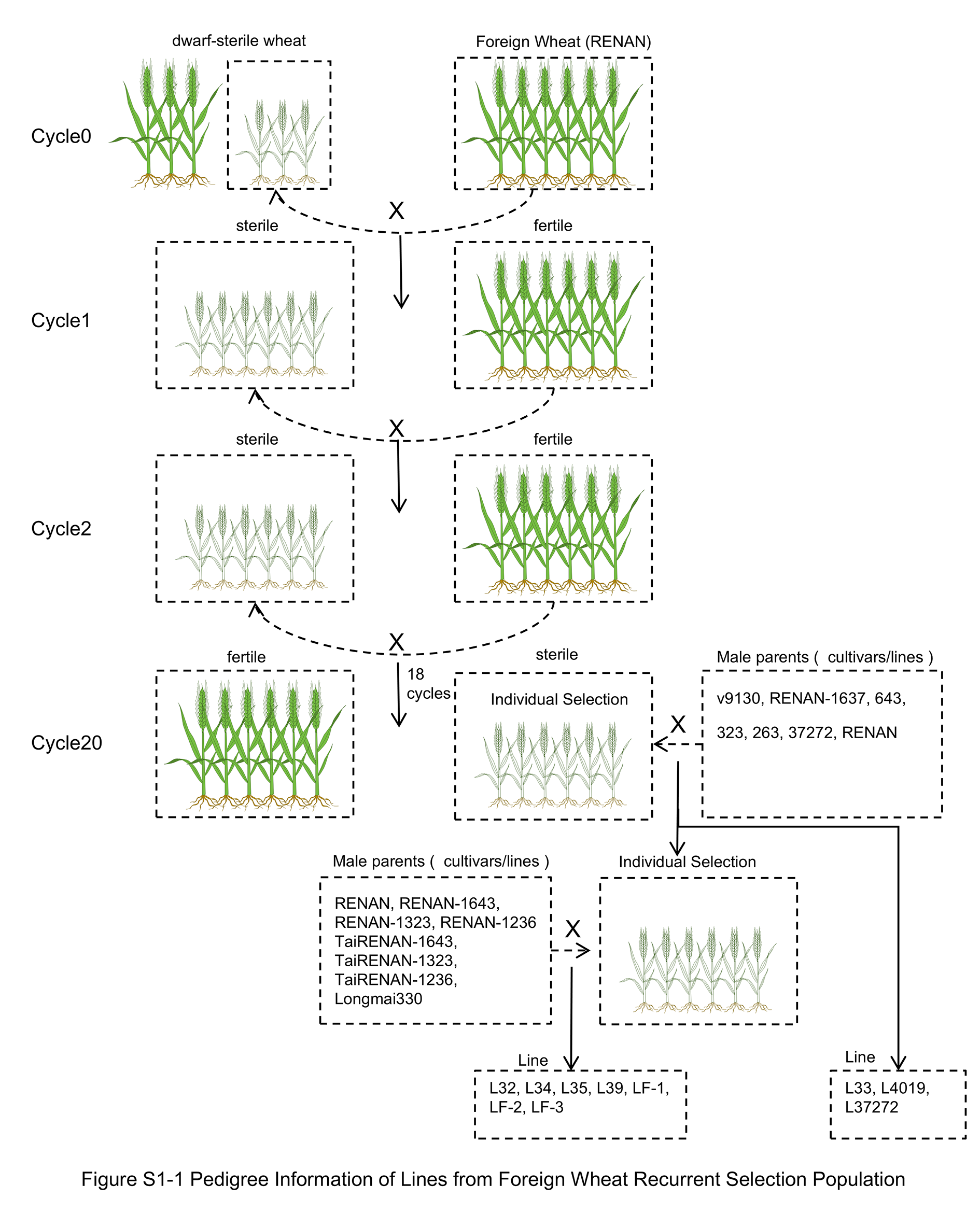

Supplement: Supplementary Figure 1 — Pedigree Information of Lines from Foreign Wheat, Spelt, Tibetan semi-wild wheat, and Spike-branched wheat Recurrent Selection Populations. [file Image1.tif]

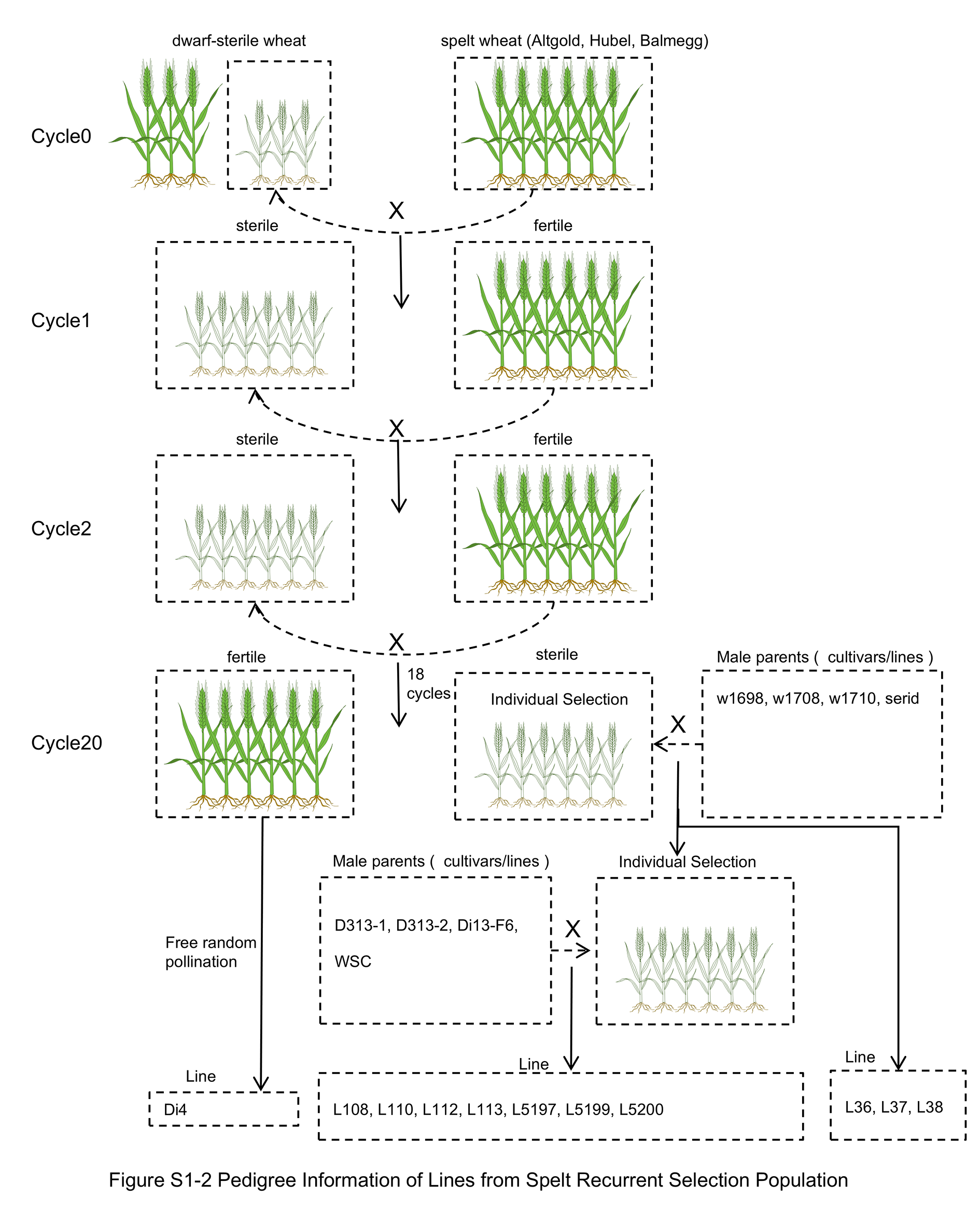

Supplement: Supplementary Figure 2 — Phenotypic Distribution of Six Traits in Populations Derived from Different Cycles of Recurrent Selection. [file Image2.tif]

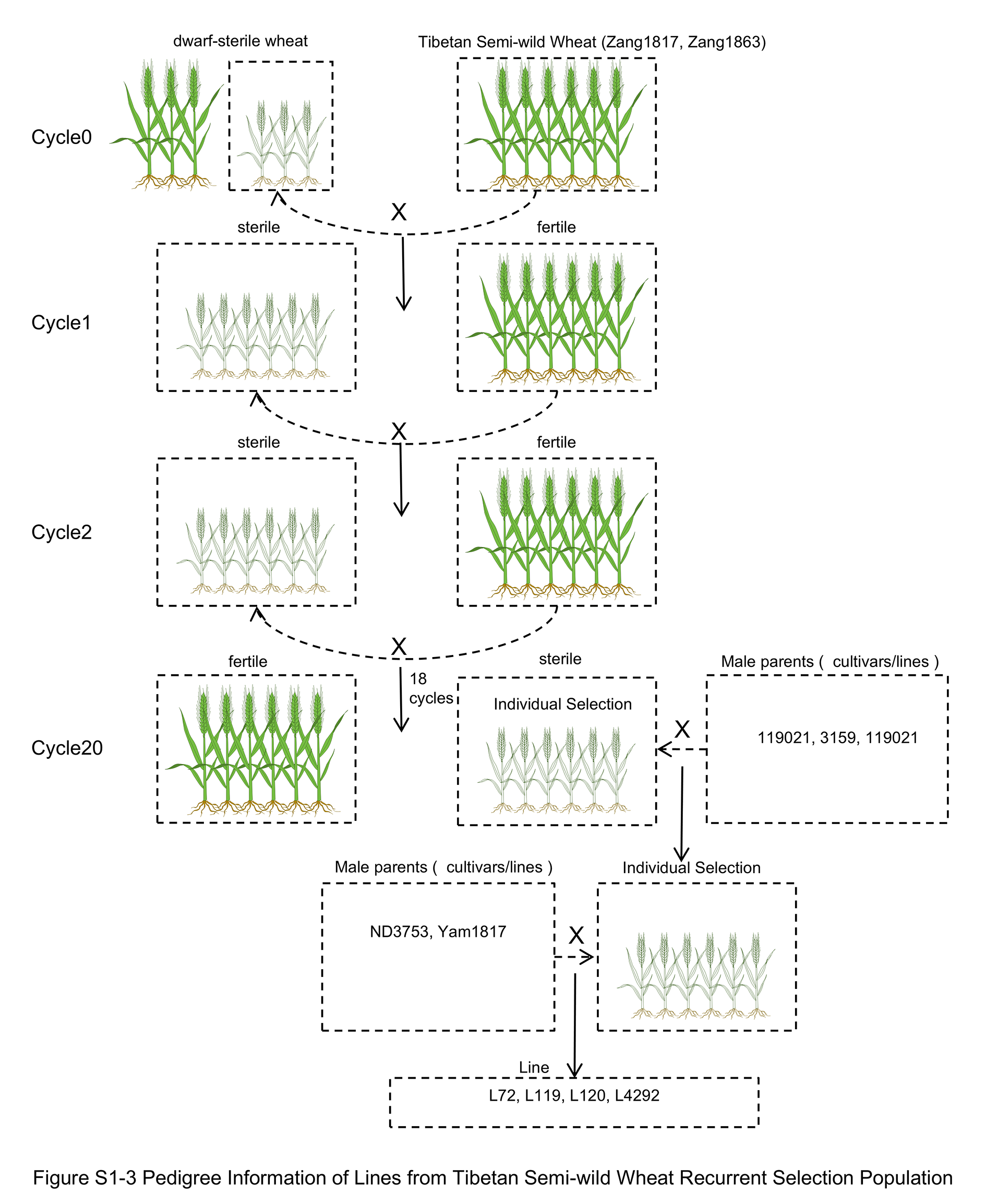

Supplement: Supplementary Figure 3 — Distribution of Markers on Each Chromosome. [file Image3.tif]

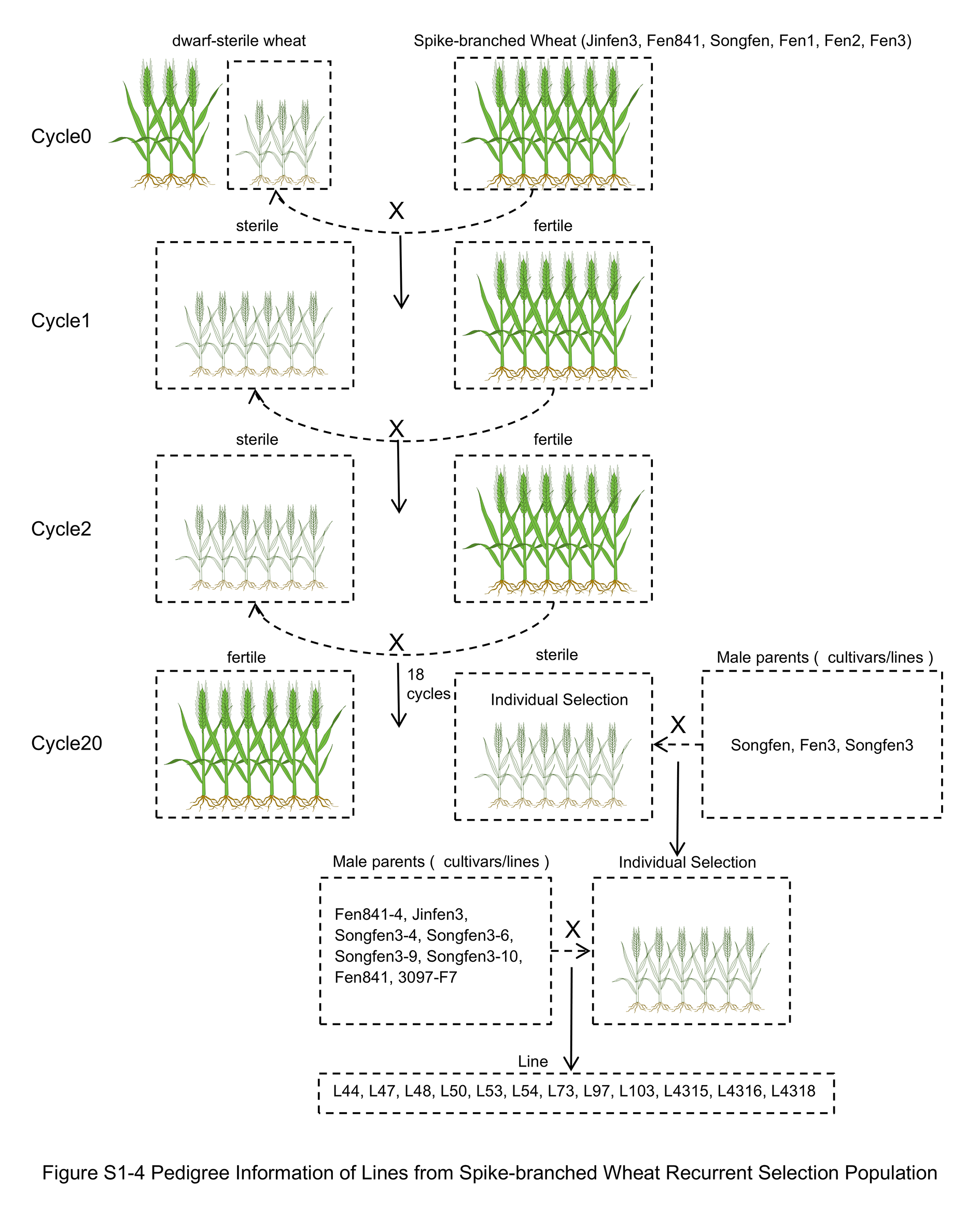

Supplement: Supplementary Figure 4 — (PCA) of 214 Wheat Lines/Cultivars. [file Image4.tif]

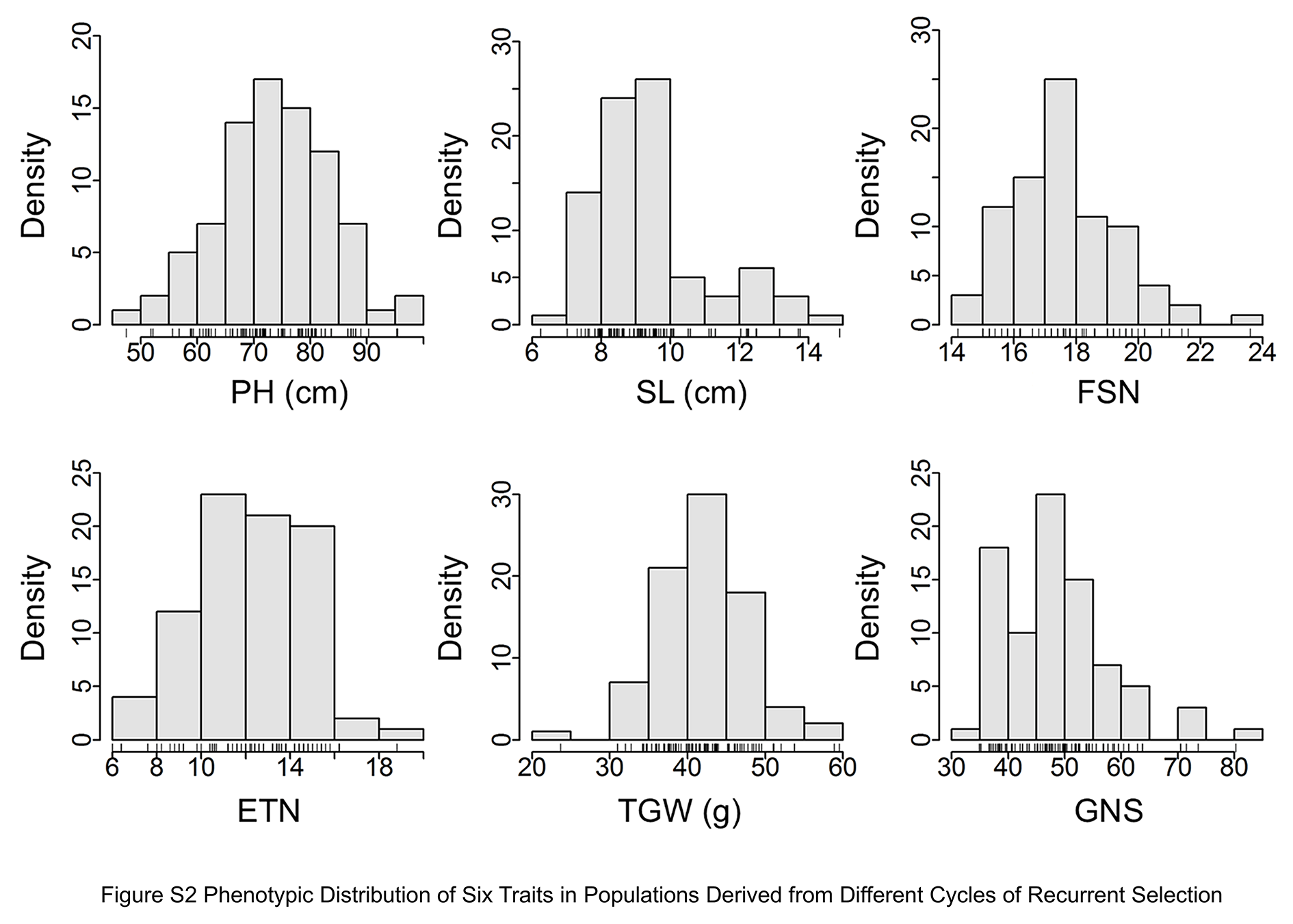

Supplement: Supplementary Figure 5 — Clustering Analysis of 214 Wheat Lines/Cultivars. [file Image5.tif]

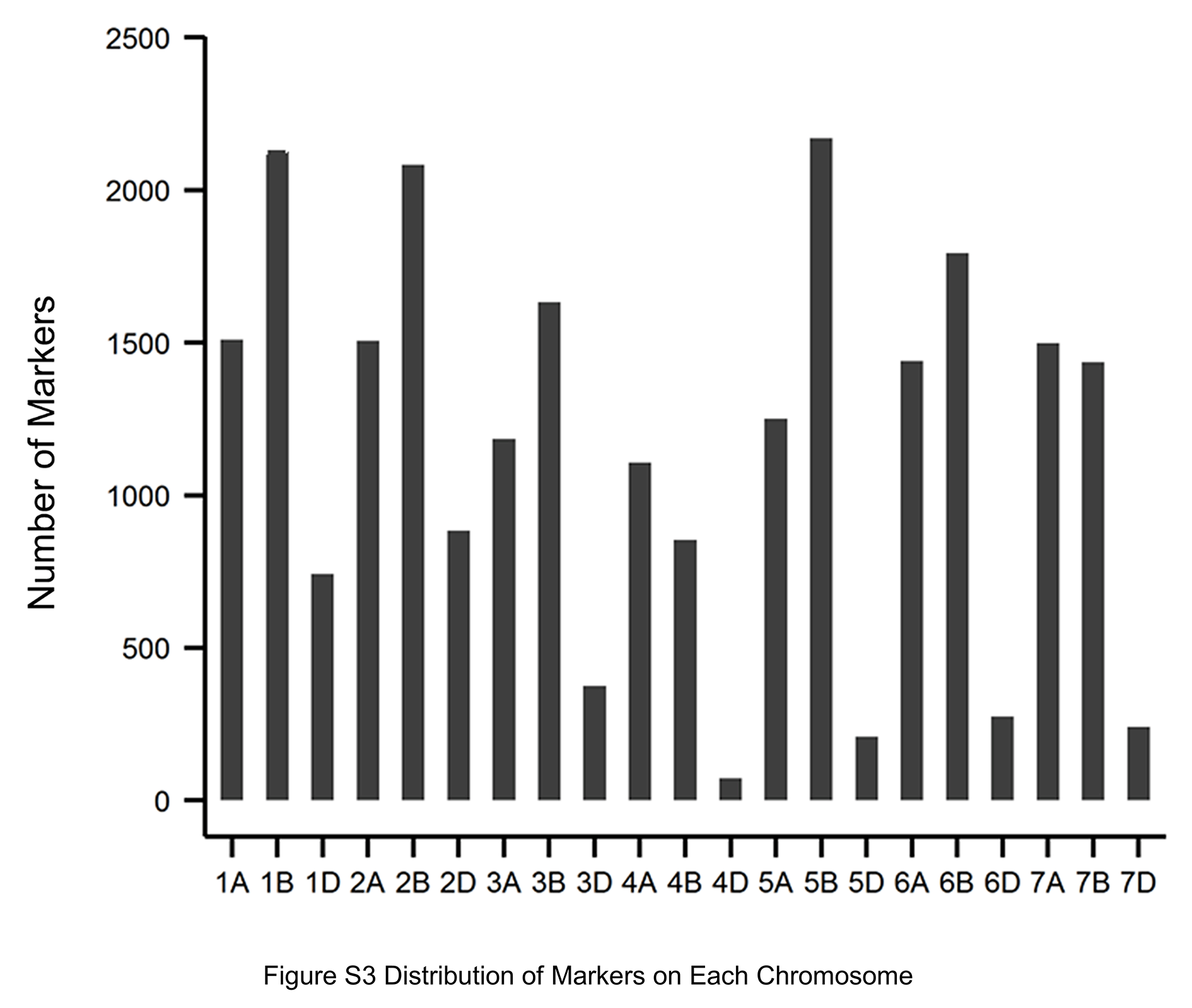

Supplement: Supplementary Figure 6 — Kinship Matrix of 214 Wheat Lines/Cultivars. [file Image6.tif]

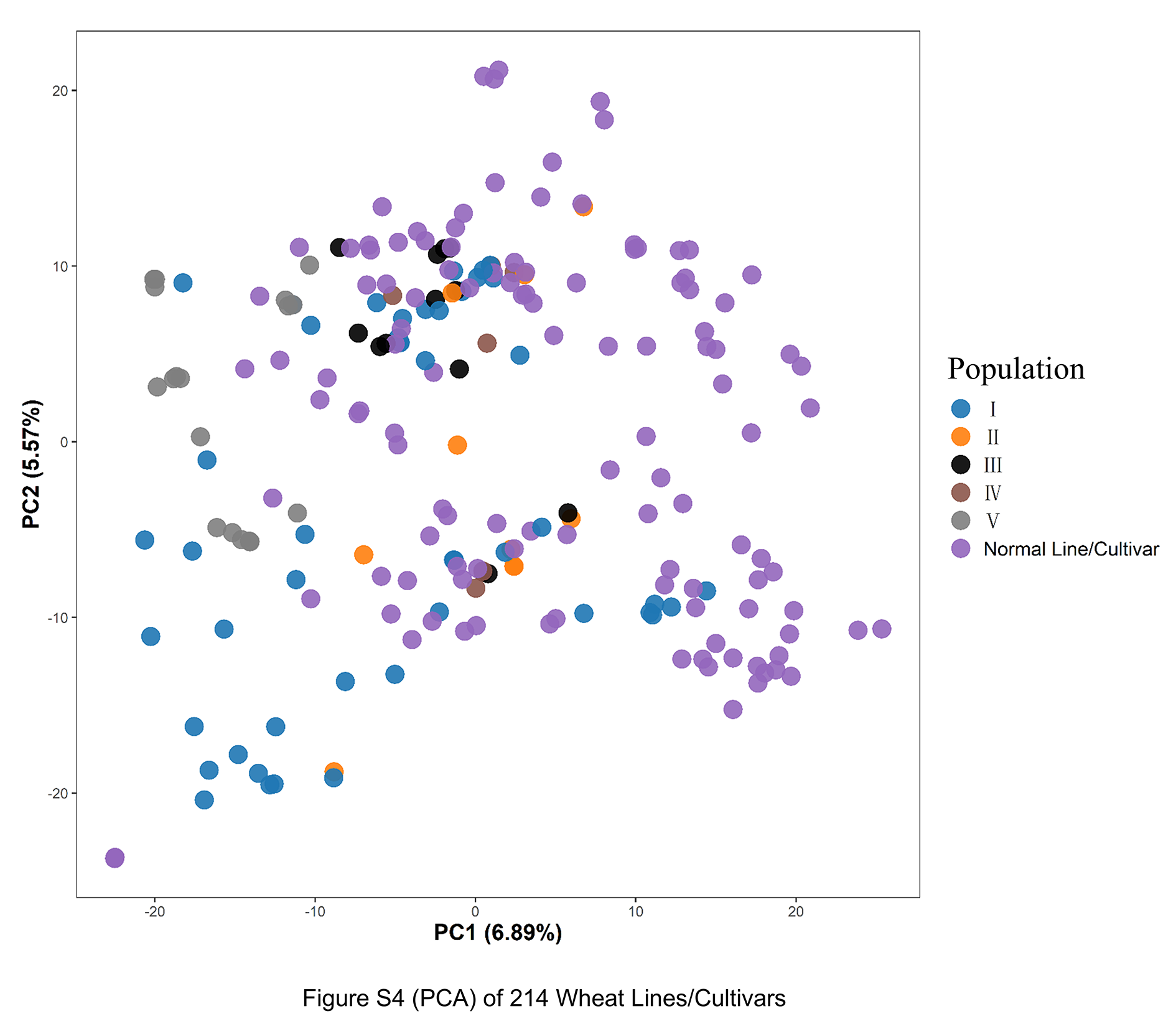

Supplement: Supplementary file 8 [file Image7.tif]

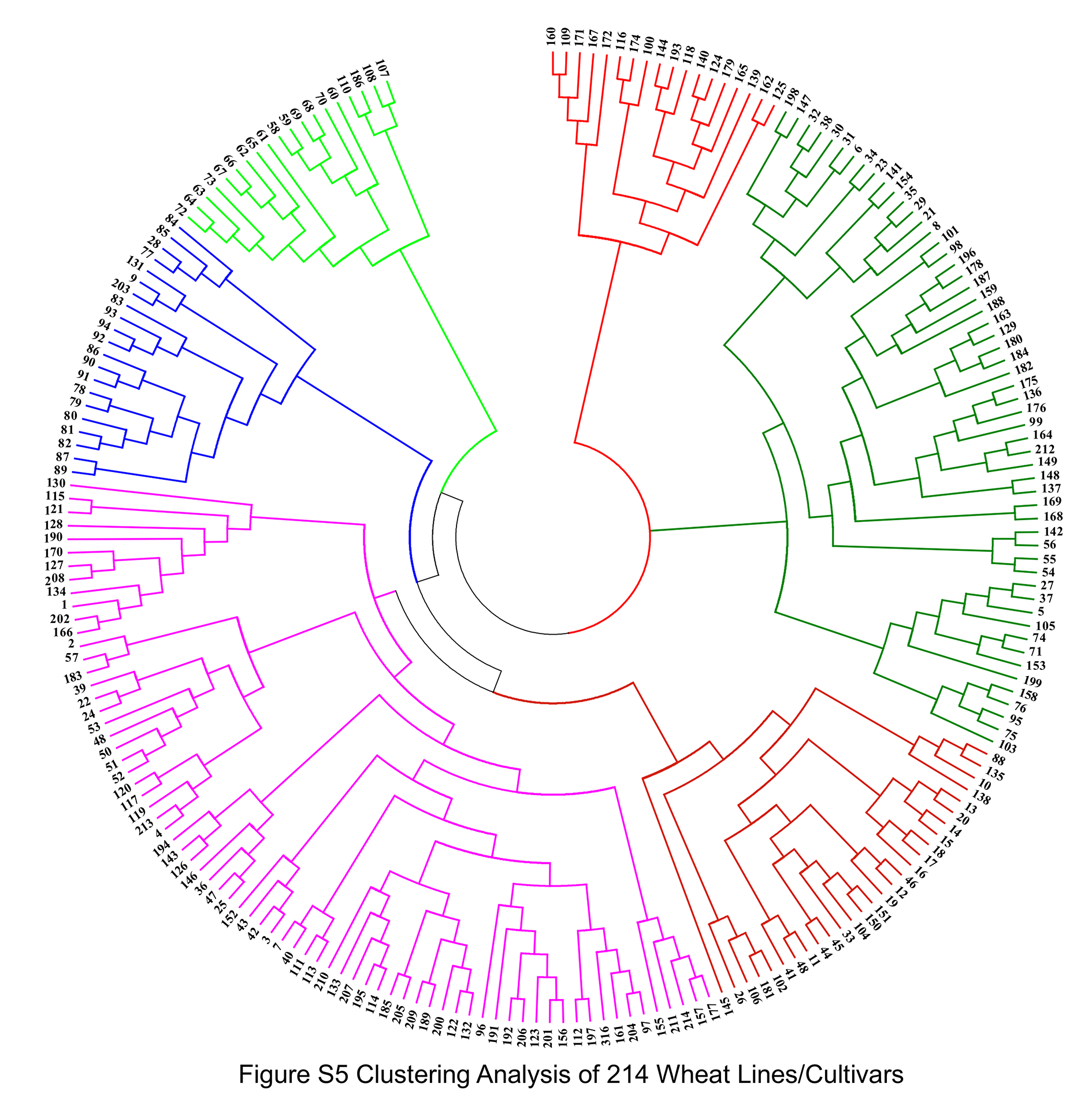

Supplement: Supplementary file 9 [file Image8.tif]

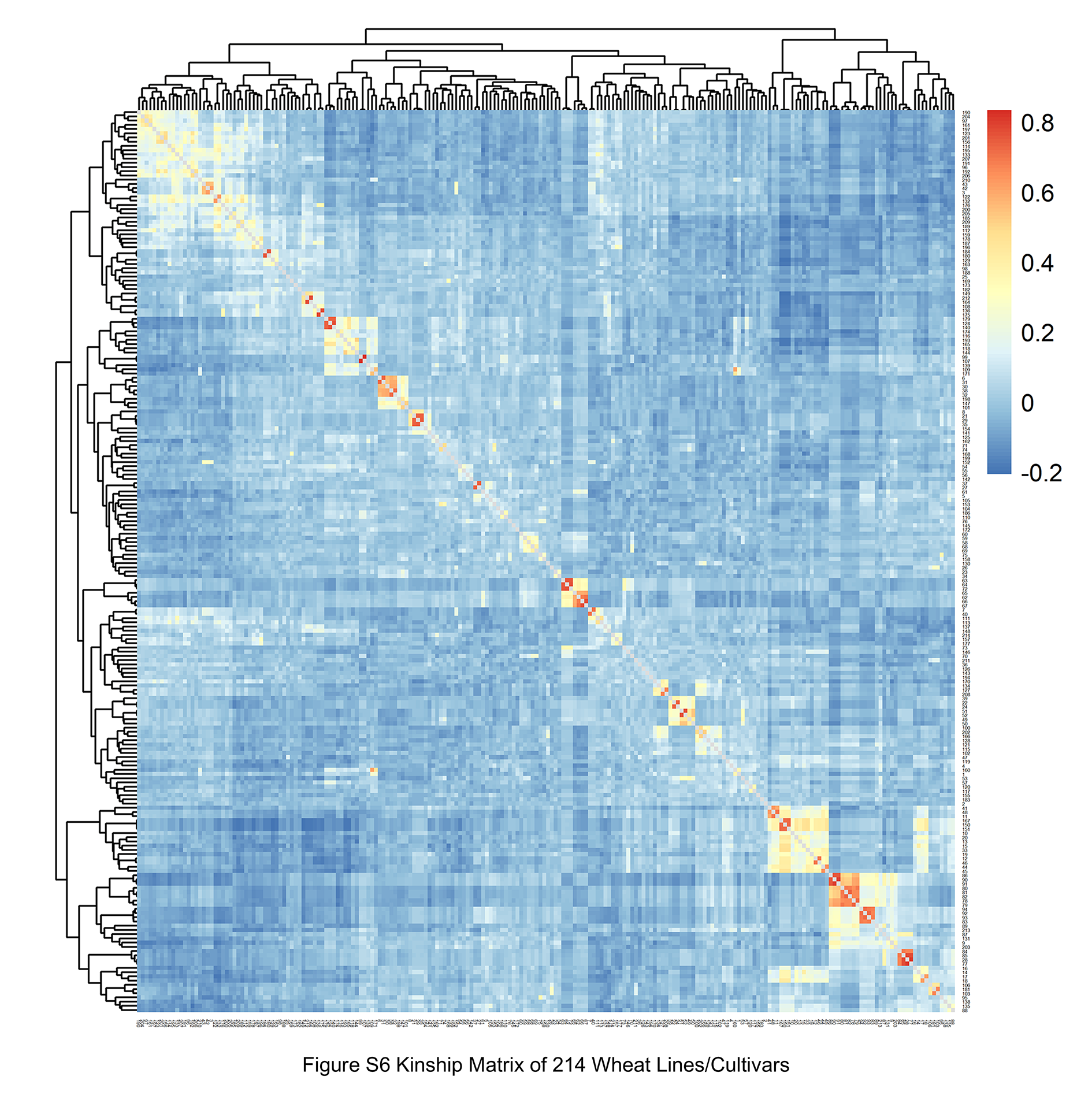

Supplement: Supplementary file 10 [file Image9.tif]
